# A non-randomised controlled study of the missing link person-centred care transition support intervention after stroke or TIA

**DOI:** 10.1038/s41598-026-45766-w

**Published:** 2026-03-24

**Authors:** Andrea Hess Engström, Ann Charlotte Laska, Maria Flink, Mihaela Oana Romanitan, Lena von Koch, Charlotte Ytterberg, Sebastian Lindblom

**Affiliations:** 1 Department of Neurobiology, Care Sciences and Society, Karolinska Institutet, Stockholm, Sweden; 2 Department of Clinical Sciences, Karolinska Institute Danderyd Hospital, Stockholm, Sweden; 3 Women’s Health and Allied Health Professionals Theme, Karolinska University Hospital, Stockholm, Sweden; 4 Research and Development Unit for Older Persons, Region Stockholm, Sweden; 5 NeuroCardioMetabol Research Group, Department of Clinical Science and Education, Karolinska Institute Södersjukhuset, Stockholm, Sweden; 6 Theme of Heart & Vascular and Neuro, Karolinska University Hospital, Stockholm, Sweden; 7 Division of Physiotherapy, School of Health, Care and Social Welfare, Mälardalen University, Västerås, Sweden; 8 Karolinska Institutet, NVS, Fack 23100, 141 83 Huddinge, Sweden

**Keywords:** Care coordination, Teach back method, Continuity of care, Patient-centred communication, Health literacy, Diseases, Health care, Medical research, Neurology, Neuroscience

## Abstract

The transition from hospital to home after stroke or transient ischaemic attack (TIA) is a vulnerable phase marked by gaps in coordination, communication and patient preparedness. This study evaluated the effectiveness of a multicomponent care transition support on perceived quality of care transition (QCT) amongst people with stroke or TIA one week post-discharge. This non-randomised controlled study was conducted in three Swedish hospitals. Adult people with stroke or TIA discharged to home and referred for home-based neurorehabilitation were eligible. Data were collected during hospitalisation and at a blinded one-week follow-up. Primary outcome was perceived QCT, assessed with the Care Transition Measure. Secondary outcomes included health literacy, medication adherence and perception of received care. In total, 163 participants completed follow-up. The intervention group reported higher perceived QCT than controls (*p* = 0.002), which remained significant after adjustments (β = 6.34; 95% CI 0.56–12.12; *p* = 0.03). The intervention group reported greater confidence in managing health and better understanding of medications. Health literacy was higher in the intervention group, with no between-group differences in medication adherence or perception of received care. A multicomponent care transition support intervention may improve perceived QCT early after discharge in people with stroke or TIA.

**Supplementary Information:**

The online version contains supplementary material available at 10.1038/s41598-026-45766-w.

## Introduction

In Sweden, acute stroke care is considered efficient^1^ with short hospital stays and subsequent follow-up care and/or rehabilitation in primary or secondary care. The sudden onset of stroke or transient ischaemic attack (TIA), combined with common consequences such as cognitive impairment, fatigue and depression^2,3^, often leaves patients and their families insufficiently prepared for coming home. The short hospital stay further limits opportunities for participation in discharge planning^4,5^, leaving patients to manage a complex, new situation on their own^6^. Although Swedish legislation assigns healthcare providers the responsibility for coordinating care, much of the burden still falls on patients and their families^7^.

There are few established programmes designed to strengthen self-management of secondary prevention after discharge^8–10^. Secondary prevention includes medical treatment and health-related behavioural changes^11^, requiring knowledge and motivation^12^ as well as health literacy. Health literacy—the ability to access, understand, appraise and apply health information to make informed decisions^13^—is associated with better function and overall health^14^, less depression, enhanced perceived stroke recovery and increased participation^15^. However, 30 days post-stroke, only 67% of patients were engaged in all recommended post-discharge secondary preventive behaviours^16^. This suggests that gaps in coordination and support may hinder patients’ ability to maintain secondary prevention practices.

Care transitions are more effective when services are integrated and well-coordinated^5,17^. Involving people with stroke in discharge planning has been shown to facilitate more tailored and person-centred services, empower patients, enhance their ability to navigate healthcare systems and foster self-management, thereby promoting recovery after stroke^17,18^. Previous studies indicate that strategies aimed at improving the quality of care transitions can enhance quality of life, improve performance of activities of daily living and reduce depression amongst people with stroke^19,20^. However, these findings largely stem from studies conducted outside the Nordic context, where care pathways, follow-up routines and access to rehabilitation services may differ substantially from those in Sweden. Moreover, methodological limitations and heterogeneity in intervention components make it difficult to draw firm conclusions. Although person-centred care is widely advocated, its application in discharge processes is often implicit and inconsistently implemented. Therefore, the person-centred *Missing Link* intervention was developed, aimed at improving quality of care transitions amongst people with stroke or TIA^21,22^. Health literacy for self-management of secondary prevention was explored as a secondary outcome, based on the assumption that improved person-centred discharge processes may strengthen individuals’ understanding of their condition and their capacity to manage secondary prevention. Following feasibility testing^21,23^, the intervention was scaled up. The aim of this study was to evaluate the effectiveness of a person-centred care transition support intervention, the *Missing Link*, on the perceived quality of care transitions for people with stroke or TIA one week post-discharge.

## Methods

### Intervention

The Missing Link intervention was developed following the Medical Research Council framework for complex interventions^24^, and refined after evaluating the feasibility, fidelity and acceptability^23^. The intervention aimed at enhancing the quality of the care transition and supporting health literacy for self-management of secondary stroke prevention after hospital discharge.

Standard care consisted of routine discharge procedures, including medical assessment, verbal discharge information from the physician, a standard discharge letter, medication review, and referral to follow-up care and/or rehabilitation according to usual clinical practice. Although individual needs may be addressed in routine care, this is not systematically structured or standardized. In contrast to standard care, the Missing Link intervention added structured person-centred dialogue using the Teach-Back method, individualized written materials, informational videos, and a digital bridging meeting with the neurorehabilitation team.

This multicomponent person-centred care transition support was tailored to individual needs and included a person-centred dialogue guiding all patient–provider communication, information on post-stroke care and rehabilitation, various pedagogical methods for delivering information, and a digital bridging meeting to support the transition home. The Teach Back method, a structured technique in which patients re-explain key information in their own words, was used to facilitate communication and ensure patient understanding, with an emphasis on individual needs^25,26^(Table 1).

**Table 1:** Overview of the intervention components.

Components	Content of the intervention component
Person-centred dialogue and Teach Back Method	The dialogue focused on three elements: (1) identifying and responding to the patient’s needs, values, preferences, and expectations; (2) ensuring understanding of the condition and secondary prevention; and (3) achieving mutual agreement on the post-discharge plan. The Teach Back method was used to confirm understanding by asking patients to re-tell key information, helping identify any misunderstandings for clarification.
Pedagogical modes of information	An individualized discharge letter written by the responsible physician was provided to the patient at discharge. It included information about the diagnosis, examinations, lifestyle recommendations, driving, follow-ups, contact information, and a medication list with dosages. This information was also verbally communicated by the physician.
Video and Bridging e-meeting	Patients received recorded informational videos and written materials introducing the neurorehabilitation team, explaining follow-up and rehabilitation plans, and summarizing the patient’s condition and secondary preventive measures. A digital “bridging” meeting, arranged by allied healthcare professionals at the hospital, brought together the patient, their significant others, and the neurorehabilitation team to coordinate the care transition and address any questions.

Prior to implementation, physicians, physiotherapists, speech and language therapists, nurses and assistant nurses at Hospital A received approximately one hour of structured training on the multicomponent intervention. The training included practice, reflection, role-play with clinical scenarios, audit and feedback, and pocket reminders summarizing intervention components for all involved staff. The training sessions were repeated throughout the study to ensure that all staff would have the opportunity to receive the training. Additional sessions were provided if needed. Members of the research team were also available on site during the implementation phase to provide support and answer questions.

A detailed description of the intervention and its theoretical framework is published elsewhere^27^.

### Study design

This non-randomised controlled study of the *Missing Link* intervention was conducted in Stockholm, Sweden, in 2022–2024, following a prior feasibility study^21,23,28^. All methods were carried out in accordance with relevant guidelines and regulations. All protocols were approved by the Swedish Ethical Review Authority (2022-02105-01 and 2022-04469-02). Informed consent was obtained from all participants. The study was reported in accordance with the TREND reporting guidelines and was registered at ClinicalTrials.gov (NCT05646589, date of registration: February 12th, 2022).

### Participants

Eligible participants were people with a first-time or recurrent stroke or TIA, discharged to home from a stroke unit or a geriatric ward and referred to a neurorehabilitation team for continued rehabilitation^29^. The exclusion criterion was inability to provide informed consent.

Intervention group participants were recruited from a stroke Unit and a geriatric ward from Hospital A, and control group participants from a stroke Unit from Hospital B and a geriatric ward from Hospital C.

Control group participants received standard care transitions, including a discharge meeting with a physician, a discharge letter and electronic referrals for follow-up care and rehabilitation^29^.

### Data collection

Data were collected at baseline (during the hospital stay) and at one week, three months and 12 months post-discharge. For the purposes of this study, only the baseline data and one-week follow-up are presented. Data collectors at one week were blinded.

### Baseline data

Participants’ sociodemographic data included age, sex (men/women), nationality (Swedish/non-Swedish), educational level (up to secondary school/university), cohabiting with partner (yes/no), work status (yes, working/no), economic status (more than enough/just enough/not enough), and whether the patient had caregiver support or home care services prior to the stroke.

Medical data included type of stroke and length of hospital stay. Comorbidities were assessed using the Charlson Comorbidity Index, which classifies comorbidities by severity: no comorbidity (score 0), low comorbidity (scores 1–2) and moderate to severe comorbidity (scores > 2)^30^. Stroke severity was assessed using the 15-item National Institutes of Health Stroke Scale (NIHSS), where higher scores indicate more neurological deficits^31^. Stroke severity was categorised as minor (≤ 4), moderate (5–15), moderate to severe (16–20) and severe (21–42)^32^.

The Modified Rankin Scale (mRS) assessed the degree of disability; scores range from 0 (no disability) to 6 (death)^33^. The Barthel Index (BI) evaluated independence in personal care and mobility, with scores ranging from 0 to 100; higher scores indicate greater independence^34^. Depressive symptoms were assessed using the Patient Health Questionnaire (PHQ-2), with results dichotomised into the presence (scores ≥ 3) or absence (scores ≤ 2) of depression symptoms^35^. Cognitive function was evaluated using the short version of the Montreal Cognitive Assessment Scale (MoCA); scores range from 1 to 15 and scores < 11 indicated cognitive impairment^36^. Perceived fatigue was rated using a visual analogue scale from 0 (no fatigue) to 100 (extreme fatigue). Perceived recovery was assessed using the Stroke Impact Scale 3.0 recovery item, rated on a visual analogue scale from 0 (not recovered at all) to 100 (fully recovered)^37^.

### Outcome variables

#### Primary outcomes

Perceived quality of care transition was assessed one week post-discharge from hospital using the Swedish version of the Care Transition Measure (CTM-15). This instrument consists of 15 items rated on a four-point Likert scale, ranging from 1 (strongly disagree) to 4 (strongly agree)^38^. Scores are calculated using linear transformation to a scale ranging from 0 to 100, with higher scores indicating a higher perceived quality of the care transition^39^.

The short version of the scale, known as the CTM-3, includes items 2, 9 and 13 from the original CTM-15 and is used in clinical settings to evaluate quality of care transitions^39,40^.

#### Secondary outcomes

Health literacy was assessed using the Health Literacy Questionnaire (HLQ), which consists of nine subscales assessing aspects of health literacy^41^. Medication adherence was measured using the Medication Adherence Report Scale (MARS-5)^42^. Scores range from 5 to 25; higher scores indicate greater adherence. Person-centred care was assessed using the 21-item General Person-Centred Care Questionnaire (GPCCQ)^43^. Higher scores indicate that patients perceived the care as more person-centred. Perceptions of received rehabilitation and hospital care were assessed as two distinct variables, each measured with a single item: ‘*I have received the rehabilitation my condition* required’ and ‘*I have received the care my condition* required’. Responses were rated on a scale from 1 (agree) to 5 (do not agree at all).

### Sample size estimation

Based on a mean CTM-15 score of 72 from our previous study^23^, 70 participants were required to detect group differences (80% power, *p* = 0.05). Allowing for a 20% dropout rate, a total of 168 (84 per group) participants was targeted to ensure adequate statistical power at the final assessment.

### Statistical analysis

Descriptive statistics summarised baseline and outcome variables. Group differences were assessed with Mann–Whitney U or Chi-squared tests. Item-level CTM-15 analyses dichotomised responses into low (1–2) and high (3–4) perceived quality.

To adjust for baseline differences, a linear regression was applied with CTM-15 total scores as the dependent variable and covariates group, age, sex, education, cohabiting status, number of days in hospital before discharge, comorbidity, stroke severity, cognitive impairment, perceived recovery and depression symptoms. Model fit (R^2^) and multicollinearity were assessed. Analyses were performed using IBM SPSS Statistics, version 28.0.

## Results

**Fig. 1:**
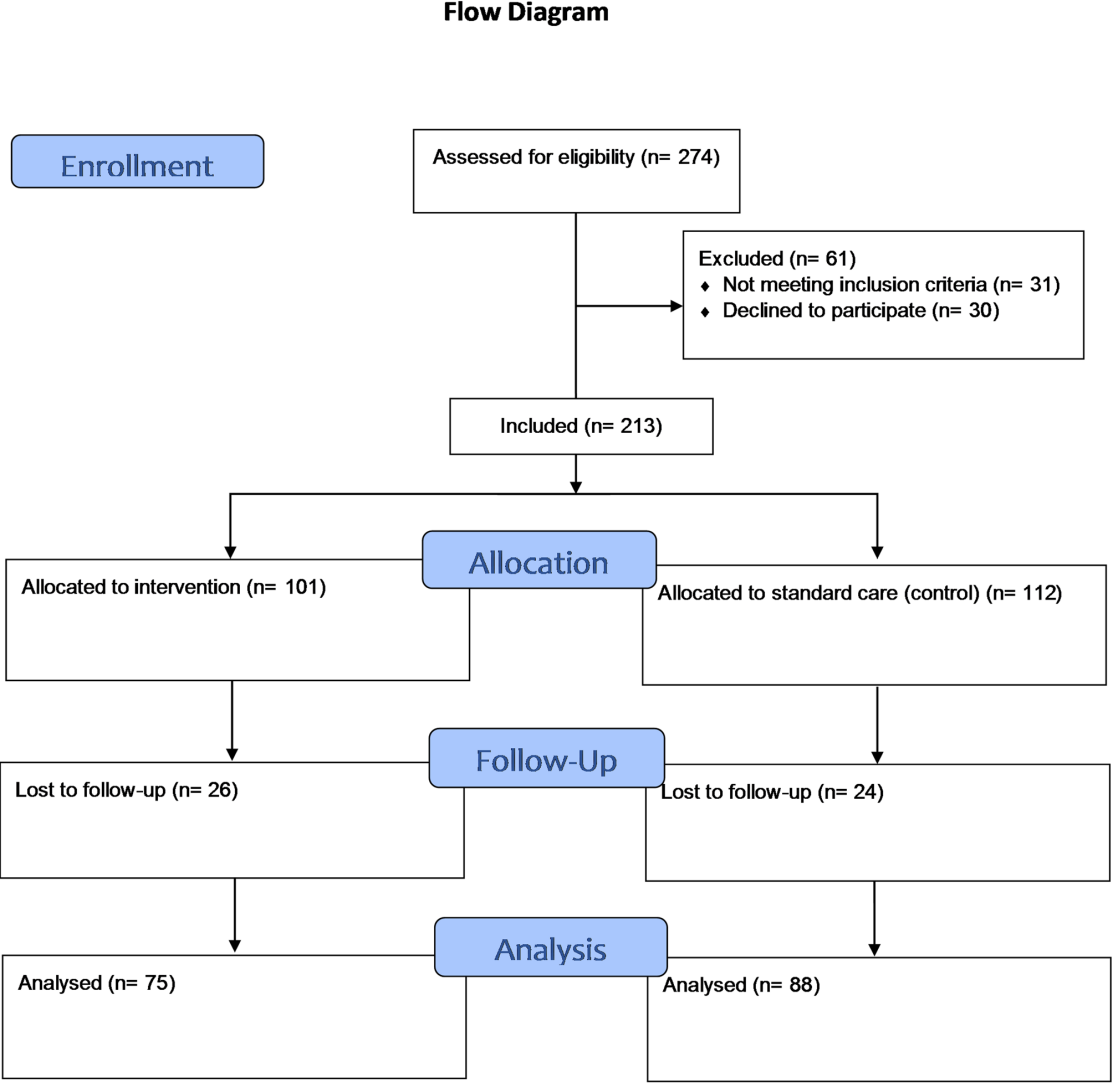
Flow diagram of participant selection and inclusion in the study.

A total of 213 participants were enrolled in the study (101 in the intervention group and 112 in the control group). A total of 163 participants completed the CTM-15 at one-week follow-up and were included in the analysis (Fig. 1).

Participant characteristics per group are presented in Table 2. At baseline, only stroke type (ischaemic vs. TIA) and number of days in the hospital differed significantly between groups. In the intervention group, a higher proportion of participants had ischaemic stroke (*p* = 0.01) and were discharged earlier than those in the control group (*p* < 0.01).

**Table 2:** Participant characteristics.

Variables^§^	Intervention *N* = 75		Control *N* = 88		
	*n* (%)	median (IQR)	*n* (%)	median (IQR)	*p*-value
Age		77 (66–84)		76 (68–80)	0.46
Sex Women	32 (43)		46 (52)		0.22
Cohabiting with partner	50 (67)		52 (60)		0.37
Nationality Swedish	63 (84)		80 (92)		0.12
Educational level Up to high school	27 (37)		37 (43)		0.48
Working	14 (19)		18 (21)		0.73
Economic status More than enough	49 (66)		56 (64)		0.76
Assistance from caregiver before stroke	20 (27)		15 (17)		0.14
Assistance from home help services before stroke	7 (9)		8 (9)		0.96
Charlson Index No comorbidity Low comorbidity Moderate/severe Comorbidity	32 (43) 30 (40) 13 (17)		31 (35) 40 (46) 17 (19)		0.39
Type of stroke^‡^ Ischemic ICH^‡‡^ TIA	52 (69) 5 (7) 18 (24)		43 (49) 1 (1) 44 (50)		0.002
Days in hospital^**^		2 (1–3)		3 (2–5)	< 0.001
mRS 0 1 2 3 4	14 (19) 13 (18) 40 (54) 6 (8) 1 (1)			16 (18) 15 (17) 43 (49) 7 (8) 7 (8)	0.49
NIHSS ≤ 4 (minor stroke)	75 (100)		88 (100)		Not tested
Barthel Index		100 (95–100)		100 (95–100)	0.39
PHQ Signs of depression	16 (22)		23 (26)		0.48
MoCA Cognitive impairment	21 (28)		29 (33)		0.49
Fatigue		48 (19–61)		50 (26–69)	0.30
Recovery after stroke		85 (63–98)		70 (50–90)	0.06

§Missing values: Cohabiting with a partner = 1; Nationality = 1; Work status = 6; Economy = 2; Education = 3; PHQ = 2; Fatigue = 3; Barthel Index = 1; mRS = 1; Recovery after stroke = 4.

‡ Difference between ischaemic stroke and TIA; ICH not tested.

‡‡ ICH = Intracerebral haemorrhage.

There were statistically significant differences between the groups in the primary outcome, perceived quality of the care transition, in favour of the intervention (Table 3).

**Table 3:** CTM one week post-discharge.

Variables	Intervention (*n* = 75) median (IQR)	Control (*n* = 88) median (IQR)	*p*-value
CTM-15 total score	73 (64–88)	67 (53–76)	0.002
CTM-15 mean	3 (3–4)	3 (3–3)	0.002
CTM 3	78 (67–89)	67 (56–83)	0.02

In the item-level analysis, Item 10 (‘When I left the hospital, I was confident that I knew what to do to manage my health’) (*p* = 0.02) and Item 15 (‘When I left the hospital, I clearly understood the possible side effects of each of my medications’) (*p* < 0.001) showed a higher proportion of participants in the intervention reporting satisfaction compared to the control group (Supplement 1). Differences between groups remained statistically significant in the linear regression model, indicating that the intervention group had higher scores in the CTM-15 one week post-discharge (Table 4). Only the covariate cohabiting status remained statistically significant in the model, indicating that living alone was associated with lower CTM-15 scores compared to cohabiting. The model explained 21% of the variance (R^2^ = 0.21). The VIFs for all predictors ranged from 1.03 to 1.42.

**Table 4:** Results of linear regression model predicting CTM-15 total score one week post-discharge.

Factor	Unstandardized Beta	95% Confidence interval	*p*-value
Group Intervention Control	6.34 Ref.	0.56–12.12 Ref.	0.03
Age	-0.11	-0.37-0.15	0.40
Sex Men Women	Ref. -4.53	Ref. -10.19-1.13	0.12
Education Up to high school College/university	Ref. 1.76	Ref. -4.14-7.66	0.56
Cohabiting with partner Yes No	-6.31 Ref.	-12.15–0.47 Ref.	0.03
Number of days in hospital	-0.40	-1.05-0.26	0.24
Charlson Comorbidity Index	-3.82	-8.06-0.42	0.08
Stroke severity (NIHSS)	1.29	-2.29-4.86	0.48
Cognitive impairment (MoCA) Yes No	0.66 Ref.	-6.24-7.56 Ref.	0.85
Perceived recovery after stroke	0.08	-0.04-0.19	0.18
Depression symptoms (PHQ) Presence of depression symptoms Absence	-3.81 Ref.	-10.27-2.65 Ref.	0.25

In the analysis of secondary outcomes, statistically significant between-group differences were observed in HLQ subscales 2–9, all in favour of the intervention group (subscale 2: *p* < 0.001; subscale 3: *p* = 0.01; subscale 4: *p* = 0.02; subscale 5: *p* = 0.04; subscale 6: *p* = 0.001; subscale 7: *p* = 0.01; subscale 8: *p* = 0.01; subscale 9: *p* = 0.01). No significant differences were found for HLQ subscale 1, MARS, GPCCQ total score, GPCCQ item 5, perception of rehabilitation or perception of care (Supplement 2).

## Discussion

This study evaluated the effectiveness of a multicomponent person-centred care transition support intervention for people with stroke or TIA on perceived quality of care transitions one week after hospital discharge. The intervention group reported higher perceived quality of the care transition compared to those in the control group, even after controlling for potential confounding factors. Evaluating the perceived quality per item of the CTM-15 revealed significant differences for Item 10 (‘I knew what to do to manage my health’) and Item 15 (‘I understood the possible side effects of my medications’), indicating that the intervention strengthened confidence in self-management. Health literacy was also higher amongst the intervention group. No significant differences between groups were observed regarding adherence to medical treatment, patient-centred care, and perceptions of received rehabilitation and care.

The finding that the intervention group reported a higher perceived quality is consistent with previous research on other multicomponent interventions designed to improve care transition. Such interventions have been shown to enhance perceived quality of care transitions in stroke populations, even though the components of these interventions vary, for example by focusing on system-level care coordination, while other focus more on education and skill-building for patients and caregivers^44,45^. The considerable heterogeneity in intervention components and outcome measures makes it difficult to determine which elements are most effective. Therefore, while our findings align with previous research, they should be interpreted with caution.

In the present study, the intervention group reported higher health literacy one week post-discharge. Prior research has indicated that low health literacy can impede patients’ understanding of discharge information and create uncertainty regarding access to follow-up care^46^. As limited health literacy may reduce patients’ engagement in care transition planning^47^, it is possible that individuals with higher health literacy are better equipped to actively participate in their care and evaluate it more favourably. Although our results may suggest that the intervention enhanced patients’ understanding of health information, the lack of baseline measurement of health literacy limits the ability to determine whether these differences were pre-existing or intervention-related. It is also possible that interventions designed to improve quality of care transitions may have a greater impact amongst individuals with higher health literacy, who are more capable of processing and utilising the information and support provided. The higher proportion of TIA in the control group may have influenced health literacy outcomes, as differences in diagnoses and perceived seriousness of the event could affect patients’ engagement with discharge information. Although baseline clinical measures were comparable between groups, this imbalance should be considered when interpreting the findings.

Lastly, the observed differences in CTM-15 scores between participants living alone and those who were cohabiting suggest that social and household context may influence perceptions of care transitions. Social and relational support may facilitate individuals’ engagement with and adherence to care transition programs, enabling them to benefit more fully from the intervention^48^. As cohabiting status remained significant in the adjusted model, this finding indicates that household context may influence perceived quality of care transitions. However, as the present study assessed only cohabiting status and not the extent of participants’ social support, the mechanisms underlying this association remain unclear.

Overall, the findings of the present study suggest that a multicomponent intervention with a focus on supporting patients’ understanding of information can enhance the perceived quality of care transitions. Assessing health literacy may therefore be useful for identifying patients in need of additional support and for guiding resource allocation more effectively^49^. Future studies should consider tailoring communication and discharge support according to patients’ individual needs and levels of health literacy. Interventions that adapt communication strategies and discharge routines to different levels of health literacy are recommended to promote patient engagement, comprehension and overall satisfaction with the care transition process.

### Strengths and limitations

This study addresses prioritised areas of transitional care research, including patients’ needs, health literacy, the exchange of information between healthcare professionals and patients, and collaboration between hospital and primary care providers^50^.

Whilst randomised controlled trials are often considered the gold standard for evaluating intervention effects, the non-randomised design of this study was deemed appropriate as randomisation would have increased the risk of contamination across study arms and been practically challenging. Allocating the intervention at the site level allowed it to be integrated into routine clinical workflow and facilitated implementation. However, site effects, including baseline differences and potential confounding, cannot be entirely ruled out. Potential differences between the intervention and control sites were therefore accounted for in the adjusted statistical models used for group comparisons. To reduce a risk of bias, data collectors at the one-week follow-up were blinded to group allocation.

Most participants in this study were Swedish-born, highly educated and had mild strokes with no or low comorbidities. These characteristics limit the generalisability of the findings to more diverse populations. The observed differences in health literacy could be influenced by sociodemographic differences between the populations served by the participating hospitals, which should be considered when interpreting the results. Moreover, as health literacy was not assessed at baseline, pre-existing differences between groups cannot be ruled out.

Whilst implementation efforts were supported throughout the study, there may be limitations regarding intervention fidelity. Although structured training sessions, repeated training opportunities, on-site support from the research team, and practical reminders were provided to enhance consistent delivery, the multicomponent nature of the intervention and its integration into routine clinical practice may have resulted in variability in how components were delivered. Aspects of fidelity will be explored in a separate process evaluation study.

## Conclusion and implications

This study indicates that a person-centred care transition support intervention may improve perceived quality of care transitions one week after hospital discharge for people with stroke or TIA. Future research should evaluate the effectiveness of this intervention in a broader and more unselected population, including patients discharged from a variety of care units and to different types of care settings. Such research should also encompass individuals with more severe stroke, varying levels of health literacy and vulnerable groups, including those living alone.

## Supplementary Information

Below is the link to the electronic supplementary material.

Supplementary Material 1

Supplementary Material 2

## Data Availability

Request for data access can be made to our Research Data Office ( mail to: rdo@ki.se) at Karolinska Institutet.
