# Supplementary material for: A non-randomised controlled study of the missing link person-centred care transition support intervention after stroke or TIA
Source: Sci Rep. 2026 Mar 24;16:9698. doi: 10.1038/s41598-026-45766-w (PMC13013583; doi:10.1038/s41598-026-45766-w)
Supplement: Supplementary file 1 — Supplementary Material 1 [file 41598_2026_45766_MOESM1_ESM.docx]

Supplemental material

Supplement 1: Difference between groups per item on the Care Transition Measure (CTM-15).

*p = 0.02, ** p <0.001
