# Supplementary material for: A non-randomised controlled study of the missing link person-centred care transition support intervention after stroke or TIA
Source: Sci Rep. 2026 Mar 24;16:9698. doi: 10.1038/s41598-026-45766-w (PMC13013583; doi:10.1038/s41598-026-45766-w)
Supplement: Supplementary file 2 — Supplementary Material 2 [file 41598_2026_45766_MOESM2_ESM.docx]

Supplemental material

Supplement 2: Secondary outcomes one week post-discharge from hospital.

| Variables | Intervention  N = 75 | Control  N = 88 | *p*-value |
| --- | --- | --- | --- |
| Health Literacy Questionnaire median (IQR)  Subscale 1: Feeling  understood and  supported by  healthcare providers  Subscale 2: Having  sufficient information  to manage my health  Subscale 3: Actively  managing my health  Subscale 4: Social  support for health  Subscale 5: Appraisal  of health information  Subscale 6: Ability to  actively engage with  healthcare providers  Subscale 7: Navigating  the healthcare system  Subscale 8: Ability to  find good health  information  Subscale 9:  Understanding health  information well  enough to know what  to do | 3 (2.38—3.63)  3 (2.75—3.50)  3 (2.80—3.60)  3.40 (3—3.80)  2.75 (2.40—3)  4 (3.60—4.60)  3.83 (3.41—4.33)  4 (3.55—4.40)  4.20 (3.80—4.80) | 2.75 (2.25—3.25)  2.75 (2.50—3)  2.90 (2.40—3.15)  3.20 (2.80—3.40)  2.40 (2—3)  3.60 (3.20—4)  3.50 (3—4)  3.80 (3.20—4.20)  4 (3.60—4.60) | 0.09  < 0.001  0.01  0.02  0.04  0.001  0.01  0.01  0.01 |
| MARS median (IQR) | 25 (24--25) | 25 (24--25) | 0.51 |
| GPCCQ total median (IQR) | 62 (50—77.50) | 60 (48—73) | 0.52 |
| GPCCQ, item 5 median (IQR) | 4 (3.50—5) | 4 (3—5) | 0.54 |
| Perception of rehabilitation n (%)  1 Agree  2  3  4  5 Do not agree at all | 48 (72)  8 (12)  6 (9)  4 (6)  1 (1) | 50 (59)  10 (12)  11 (13)  5 (6)  9 (10) | 0.06 |
| Perception of care n (%)  1 Agree  2  3  4  5 Do not agree at all | 58 (82)  7 (10)  1 (1)  3 (4)  2 (3) | 65 (77)  6 (7)  8 (9)  4 (5)  2 (2) | 0.40 |

Missing values: HLQ, subscales 1 to 5 = 2; HLQ subscales 6-9 = 3; MARS = 1; GPCCQ = 3; HLS = 7; Perception of rehabilitation = 11; Perception of care = 7.
